# Comparison of Different Methods of Education in the Adoption of Oral Health Care Knowledge

**DOI:** 10.3390/dj9100111

**Published:** 2021-09-26

**Authors:** Lidia Gavic, Martina Marcelja, Kristina Gorseta, Antonija Tadin

**Affiliations:** 1 Study of Dental Medicine, School of Medicine, University of Split, Soltanska 2, 21000 Split, Croatia; atadin@mefst.hr; 2 Dental Practice, Health Centre of Pozega-Slavonian County, Matije Gupca 10, 34000 Pozega, Croatia; martina.marcelja@gmail.com; 3 Department of Paediatric Dentistry, School of Dental Medicine, University of Zagreb, Gunduliceva 5, 10000 Zagreb, Croatia; gorseta@sfzg.hr

**Keywords:** child oral health, dental caries, dental education, prevention

## Abstract

Aim: The scope of this study was to determine if there is a critical distinction in the usage of lectures, videos, and pamphlets as educational material utilized in the adoption of oral health care knowledge. Materials and methods: Three-hundred and thirty children from ages 11 to 13 from the city of Split, Croatia completed the questionnaire on oral health care knowledge. Consequently, they were educated by randomly using a method: lecture, pamphlet, or video. Finally, after education, their knowledge was tested again. Results: Different statistical tests were used for comparison of different sets of data. The Wilcoxon signed-rank test showed a statistically significant difference (*p* ˂ 0.001) compared to the results before and after education. The Kruskal–Wallis test comparing knowledge outcomes after three different types of education: video, lecture, and pamphlet, showed a statistically significant difference in the final knowledge between groups (*p* ˂ 0.05). A pairwise comparison between different types of education showed a significant statistical difference between education conducted by pamphlet and video material (*p* = 0.003) and pamphlet and lecture (*p* = 0.006). No difference was observed between the level of knowledge acquired through video material education and lectures (*p* = 0.928). Conclusion: Videos and lectures as means of education showed equal effectiveness in the adoption of oral health care knowledge, while the pamphlet was a method that proved to be less effective.

## 1. Introduction

General oral health has many layers, which include different abilities to talk, grin, smell, taste, touch, chew, swallow, and show a variety of fillings with certainty and without pain, discomfort, and disease of the craniofacial complex [1].

Prevention plays a crucial role in dental medicine, whose main goals are promoting and preserving oral health and which focuses on modifying or eliminating the etiological factors that drive the caries process, which includes the promotion of healthy habits and education, the proper use of fluoride, and proper diet with minimal sugar intake [2,3].

The primary goal of oral health education is to provide information and to further develop knowledge to motivate individuals to adopt healthier lifestyles, while changing attitudes and habits. Education is essential in adolescent children. During adolescence, young people can learn and maintain healthy attitudes and behaviours that they can facilitate throughout life. Therefore, the school would act as the best environment where oral health prevention measures would be taught [4].

Learning is an active process and is mainly a socio-cognitive activity [5]. Furthermore, learning and teaching are continuous processes that occur simultaneously. Learning is a complex process and contains five main components: source (lecturer), receiver (students/patients), message (content/lecture), channels (lecture, pamphlet, video), and feedback (performance) [6].

Health promotion helps individuals in controlling, improving, and maintaining their health [4]. Health education and health promotion are increasingly perceived as approaches to obtain global public health goals. The main concern of health education is health behaviour. According to Cockerman, health behavior is the activity undertaken by people for the purpose of maintaining or enhancing their health, preventing health problems, or achieving a positive body image [7]. Changes in oral health behaviour are the greatest hope in reducing oral and dental diseases worldwide. Therefore, positive, informed changes in health behaviour are usually the ultimate goals of health education programs [8].

Oral health literacy is defined “as the capacity to obtain, interpret and understand basic health information and services and the competence to use such information to enhance health” [9]. Limited literacy affects health behaviours and health decisions, including preventive services, which negatively affects health outcomes. Today’s health environment is complex and requires increased patient responsibility to move through various health services systems, make health decisions, and follow therapeutic plans. Therefore, improved health literacy can lead to improved health outcomes. Still, most educational materials on oral health are written at a level beyond what most adults can understand. In addition, healthcare providers often have high expectations about the ability of their patients to understand information. Therefore, it is recommended that the level of complexity of most patient educational materials be at the level of fifth or sixth grade [10].

The decayed, missing, and filled teeth (DMFT) index represents the number of carious, extracted, and filled teeth, and, since 1938, the World Health Organization (WHO) has used the DMFT index as a relevant factor in assessing oral health. Twelve-year-olds stand out as a critical age group due to reliable and straightforward monitoring during schooling. Therefore, the World Health Organization (WHO) takes them as a global age group for monitoring oral health status [11].

Generally, in the Republic of Croatia, there has not been a study that would compare these three educational tools. The usage of these methods are widely spread in both formal and informal education. Thus, it was necessary to investigate the relationship between education tools and adopted knowledge, with the particular references to the oral health care knowledge.

## 2. Materials and Methods

The research was approved by the Ethics Committee of the Medical Faculty of the University of Split (Class: 003-08/20-03/005, Reg. No.: 2181-198-03-04-20-0014). The total number of children in the fifth or sixth grade of the primary school in in the city of Split was 3674. Therefore, within a 5% of a statistical error, a 90% confidence interval, and a 50% response distribution, the minimum of the required sample size was 253.

The purpose of the study was presented to principals of each school that participated. After their approval, the principals forwarded to parents informed consent forms to be signed. Participation in the survey was voluntary, and parents signed the informed consent forms. The research was conducted between January and March 2020 in schools in the classrooms. The inclusion criteria were children of the fifth or sixth grade of the primary school whose parents had approved their participation in the research with written consent. The exclusion criteria were children who already had some form of oral health education and children whose parents had not approved their study participation. The questionnaires were anonymous; the children in the study did not use their full name but a code consisting of an easy-to-remember word and numbers in a sequence representing their date of birth (e.g., Flower1706, Ana1210). After a thoughtful and comprehensive literature review, two experts in pediatric dentistry designed the questionnaire for this study. The questionnaire was in the Croatian language and was pre-tested and validated via a pilot study. The pilot study participants were ten 5th grade and ten 6th grade children from the city of Makarska who reported no struggles in understanding and completing the questionnaire. The participants of the pilot study were excluded from the main study analyses. Cronbach’s alpha coefficient of internal consistency for this questionnaire was 0.846, which implied excellent reliability [12].

The first part of the questionnaire consisted of demographic data (gender, age) and questions related to the habits and attitudes regarding the oral hygiene. The second part of the questionnaire was related to general knowledge on oral health. It was composed of 17 questions with answers offered on a five-level-Likert scale from “I don’t agree at all”, “I don’t agree”, “I don’t know”, “I agree”, and “I completely agree.”

After completing the questionnaire, the children were provided education using randomly selected methods (lecture, pamphlet, or video).

All educational materials contained identical information needed to complete the questionnaire about general knowledge on oral health. The colour-printed pamphlet consisted four A2 pages. The children had a possibility to study it for 20 min and were able to take it home. The presentation consisted of 21 PowerPoint slides. The video contained images and text, which were accompanied by a combination of a background voice and a quiet melody. It lasted for 6 min and 10 s. The exact text and age-appropriate images were included in all three education materials. After education, the children filled the questionnaire about general knowledge on oral health.

## 3. Statistical Analysis

All correctly completed questionnaires were entered into the Microsoft Excel 2007 (Microsoft Corporation, Redmond, WA, USA) program and, upon completion of the research, statistically processed using the SPSS software package (IBM Corp., Armonk, NY, USA). Descriptive statistics were used to determine the basic statistical parameters (mean values, standard deviation, median, and minimum and maximum values). The Kolmogorov–Smirnov test checked the distribution of knowledge results before and after the education. The obtained results were compared with the Wilcoxon signed rank test. The Kruskal–Wallis test was used to compare knowledge results after three different types of education (video, lecture, and pamphlet). The significance level *p* < 0.05 was used in all analyses.

## 4. Results

The study included 330 children: 173 (52.42%) girls and 154 (46.36%) boys, aged 11 to 13 years, with an average age of 11.45 ± 0.56. The first part of the questionnaire referred to the habits and attitudes of children about maintaining oral hygiene.

When asked how many times they brushed their teeth, the most significant number of 211 children (63.94%) answered twice a day, followed by the answers: “three times a day” by 62 respondents (18.79%), “once a day” by 46 respondents (13.94%), and “four times a day” by 11 respondents (3.33%). The length of brushing the teeth ranged from 0 to 15 min, averaging 3.29 ± 1.94 min. Most of the respondents (*n* = 257, 77.88%) knew that there are different levels of hardness of the brushes, and 82.49% of them believed that it is best to use a medium-hard brush. Others believe that the best brush is the soft one (31.12%) or the hard one (7.78%). Although 98.78% of children have a toothbrush, four children in the research stated that they do not have their own brush. After brushing their teeth in the evening, 44.24% of children stated that they ate or drank something other than water.

Only 6.70% of children in this study do not eat sweets. However, other children eat sweets daily (46.02% once a day, 37.58% twice a day, or 9.70% three or more times a day).

Most children (81.21%) in this study visited the dentist most recently a few months ago. Furthermore, 12.42% of children were the last at the dentist a year ago, and 3.33% two years ago. On the other hand, six children (1.82%) stated that they had never been to a dentist.

After the evening teeth wash, 53 children (44.24%) stated eating or drinking something other than water. When asked what they eat and drink after evening brushing, among other things, the following answers were found: apples, bananas, snacks, sandwiches, biscuits, bread with Nutella, fruit, cereal, yoghurt, milk, juices, teas, chips, etc.

The results of the general knowledge on oral health before and after education are presented in the Table 1.

The Kolmogorov–Smirnov test determined an incorrect distribution of knowledge results (*p* ˂ 0.001). The Wilcoxon signed rank test showed a statistically significant difference (*p* ˂ 0.001) in the comparison of results before and after education.

The Kruskal–Wallis test, which compared the outcomes after three different types of education (video, lecture, and pamphlet), showed a statistically significant difference in the knowledge between groups (*p* = 0.004). Pairwise comparison between different types of education showed a significant statistical difference between education given through the pamphlet and video material (*p* = 0.002) and pamphlet and lecture material (*p* = 0.016). No difference was observed between the level of knowledge acquired through the video material education and lectures (*p* = 0.984).

**Figure 1:**
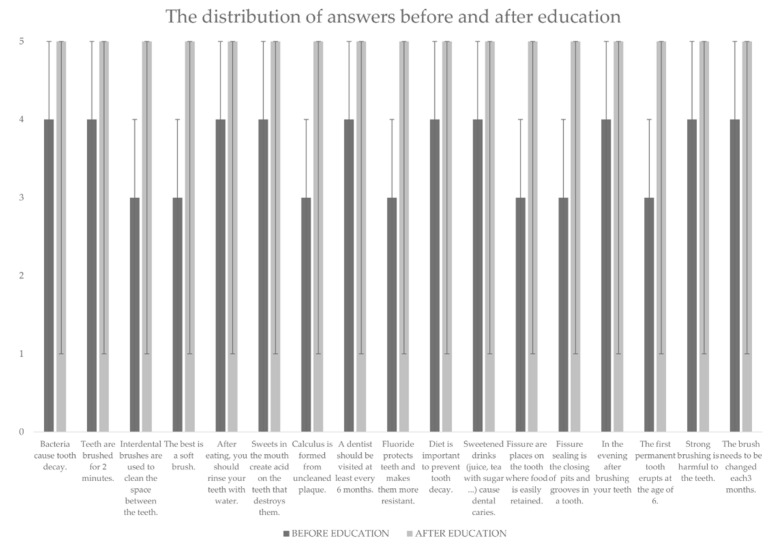
The distribution of answers before and after education. (Statistical significance was observed on each question).

According to the Wilcoxon signed rank test, there was a statistically significant difference in the distribution of answers before and after education to each question (Figure 1). However, comparing the distribution of answers after education, observing three forms of education, a significant difference was observed on only four questions (Table 2).

## 5. Discussion

This intervention study aimed to examine the effectiveness of different methods of teaching on children’s knowledge of oral health and to determine whether there is a difference in the types of teaching techniques. After the education, a significant improvement in knowledge was observed (*p* ˂ 0.001). However, the knowledge after the pamphlet education proved to be weaker than the education in videos and lectures. Thus, the null hypothesis was rejected.

Similar results were obtained in the study conducted by Ahmad et al. comparing videos and pamphlets on educating the public about the environment. Namely, most of the participants enjoyed watching the video but were not satisfied with studying the pamphlet [13]. In the mentioned study, the authors believed that video had proven to be a more helpful material due to the attractiveness obtained by adding background music, animations, narration, and various visual representations [13]. Lectures have been the most common form of teaching and learning for a long time. A well-organized lecture is still one of the best approaches to integrate and introduce data from different sources. In addition, the lecture has its advantages, such as direct contact between the lecturer and the listener [6]. Lecturers have immediate feedback during the lecture and can re-explain each part if there is a need for it. In addition, the lecturer can motivate the listeners with questions and the way they speak, while, at the same time, listeners can ask questions related to the topic [5].

Although the pamphlet showed less satisfactory results in the study, it is still effective. In health promotion, pamphlets have become a viral and widely used means of educating the public. The advantages of the pamphlet as an educational tool are its economic convenience, ease of circulation, and easy display and distribution. There is also the possibility of re-reading material whenever the reader wants or when information is needed. Therefore, they are one of the media of choice for spreading knowledge to the general public [13,14]. Unfortunately, pamphlets can sometimes be incomprehensible, leaving no impact on the reader and failing to serve as an effective educational tool. The main problem contributing to the inefficiency of pamphlets is the level of linguistic expressions used [14,15].

Observing the distribution of responses after each type of education, a significant difference was noticed in only five of the seventeen statements (Table 2).

Those statements contained the terms “plaque”, “fissure”, and “fluoride”, which are not original Croatian words but, instead, are words taken from Latin [16]. Additionally, “fluoride” is a chemical element, and teaching chemistry in Croatia starts in 7th grade. Therefore, it is possible that unknown words remained the least noticed when reading the pamphlet.

The World Health Organization (WHO) recommends establishing a comprehensive information system at the national level with the aim of continuous monitoring of oral health. Data on the oral health of the population and the prevalence of risk factors are essential for planning interventions by health authorities and for understanding disease trends over time [17]. According to the latest data from 2015, the DMFT index of 12-year-olds in Croatia was high compared to other European countries, indicating inferior results in the oral health of children in Croatia [18]. The mentioned age of twelve represents a reliable age for epidemiological research on oral health and child development in general. Namely, apart from the fact that most permanent teeth have grown in most children at that age, except the third molar, in many countries, this is the last year when children can be monitored through the school system. For these reasons, 12 years was chosen as the global indicator for international comparisons and surveillance of the disease [17]. Based on everything mentioned, children of the 5th and 6th grades of primary school were included.

In this research, children’s habits of oral hygiene were observed. The highest percentage of children (63.94%) reported brushing their teeth twice a day. That number is much higher than the results obtained in a study by Mishra et al. in Lucknow, India, where only 18.1% brushed their teeth twice a day [19]. In the guidelines for oral hygiene, the World Dental Federation (FDI) recommends brushing twice a day, for a minimum of two minutes [2]. In this study, the average length of brushing was 3.29 ± 1.94 min.

Out of the total number of our respondents, only 6.70% do not eat sweets at all. However, other children (93.3%) eat them every day, of which as many as 53.98% eat them several times a day. Therefore, in this study, the consumption of sweets was much higher than those obtained in Mishra et al., where 50% of children stated a daily consumption and only 4.8% stated that it was several times a day [19].

The information that diet is essential for preventing dental caries was not known to 33.93% of children surveyed. Moreover, only 23.03% of children completely agreed that sweetened beverages cause dental caries. By contrast, in the study of Blaggana et al., 92.7% of respondents knew that sweets affected oral health [20]. However, after the education, the results improved, and as many as 66.36% of children completely agreed that the consumption of sugary drinks causes tooth decay.

Most children, 268 of them (81.21%), stated that they last visited a dentist a few months ago. The most recommended period between two visits to the dentist is six months [21,22]. Before education, only 31.81% of children fully agreed that a dentist should be visited every six months. After education, 74.84% completely agreed with the mentioned statement.

Six children (1.82%) had never been to a dentist. Considering the National Program “Dental Passport”, this number should be lower because children are referred to a dentist for enrolling in the 1st grade of elementary school [23]. However, when we compare the data with the 2016 survey by Blaggana et al. in Chandigarh, we have satisfactory results. Namely, in the aforementioned study, only 24.9% of children had been to a dentist in the past six months and 25.4% of children had never been to a dentist [20].

Although 98.78% of children have their toothbrushes, unfortunately, four children stated in the study that they do not have them. This result correlates with the research of Zhu et al., where 1.5% of children say that they do not have their toothbrushes [24].

The difference in the hardness of the brush fibers was not known by 22.12% of children. As many as 82.49% of them mistakenly thought that it is best to use a medium-hard brush. Soft toothbrushes are recommended to reduce plaque while minimizing damage to dental tissues [25].

To improve the habits of oral hygiene in children, and thus with age in adults, high-quality projects are needed to promote oral health and caries prevention. Therefore, it is imperative to encourage the revitalization of preventive dental health care in preschool and school ages. Data on the DMFT index in Croatia show that dental caries is still a public health problem, so education and further motivation of individuals are crucial to enable changes and to emphasize the importance of preventive action [11]. This study showed that all three forms of education are effective, and despite minor differences in effectiveness, children improved their knowledge of oral health. There was no statistically significant difference in children’s knowledge after education by lecture and video. However, after the education through the pamphlets, the children had lower knowledge compared to the other two types of education.

This study is the first of this kind in the Republic of Croatia, which can be the basis for further studies. In addition, based on the results of this study, the national guidelines for oral health care education could be set. However, this study has certain limitations. The whole process was time-limited in a 45-min lesson, so some answers could have been provided quickly without paying attention to the wording of the question and could have not revealed the actual attitude. Furthermore, there is a possibility that some answers were intentionally misleading. For example, the respondent jokester is a common occurrence at the adolescent age [26].

The original idea of the study was to examine, also, the children’s level of knowledge three months after the education. However, the situation caused by the COVID-19 virus disrupted the research. Therefore, it is planned to include more children from all over Croatia in the future and monitor the adoption of oral health care knowledge over a long period after the education. However, while education cannot be held in-person at the time of the pandemic, we can conclude that video education is just as effective.

## Figures and Tables

**Table 1:** The results of the general knowledge on oral health before and after education.

Knowledge Score	N	Mean (SD)	Minimum	Maximum	Median	
Before education	330	59.71 (5.47)	38	59	60	*p* ˂ 0.001 *
After education	330	77.76 (7.01)	51	85	80	*p* = 0.004 **
Pamphlet	100	76.05 (7.13)	52	85	77	
Video	90	78.47 (6.58)	62	85	81	
Lecture	140	78.52 (7.04)	51	85	81	

* The Wilcoxon signed-rank test. ** The Kruskal–Wallis test.

**Table 2:** The distribution of answers after education, observing three forms of teaching (video, lecture, and pamphlet).

		Video	Lecture	Pamphlet	Kruskal–Wallis Test
Bacteria cause tooth decay.	Median	5	5	5	0.012
	Minimum	1	1	2	
	Maximum	5	5	5	
Teeth are brushed for 2 min.	Median	5	5	5	0.386
	Minimum	4	1	1	
	Maximum	5	5	5	
Interdental brushes are used to clean the space between the teeth.	Median	5	5	5	0.125
	Minimum	3	1	1	
	Maximum	5	5	5	
The best is a soft brush.	Median	5	5	5	0.913
	Minimum	2	2	2	
	Maximum	5	5	5	
After eating, you should brush your teeth with water.	Median	5	5	5	0.434
	Minimum	1	1	1	
	Maximum	5	5	5	
Sweets in the mouth create acid on the teeth that destroys them.	Median	5	5	5	0.792
	Minimum	4	1	4	
	Maximum	5	5	5	
Calculus is formed from uncleaned plaque.	Median	5	5	5	0.004
	Minimum	3	1	1	
	Maximum	5	5	5	
A dentist should be visited at least every 6 months.	Median	5	5	5	0.513
	Minimum	1	2	1	
	Maximum	5	5	5	
Fluoride protects teeth and makes them more resistant.	Median	5	5	5	0.021
	Minimum	1	3	1	
	Maximum	5	5	5	
Diet is important to prevent tooth decay.	Median	5	5	5	0.508
	Minimum	2	1	1	
	Maximum	5	5	5	
Sweetened drinks (juice, tea with sugar, etc.) cause dental caries.	Median	5	5	5	0.965
	Minimum	1	1	1	
	Maximum	5	5	5	
Fissure are places on the tooth where food is easily retained.	Median	5	5	5	0.059
	Minimum	1	1	2	
	Maximum	5	5	5	
Fissure sealing is the closing of pits and grooves in a tooth.	Median	5	5	4	0.001
	Minimum	1	2	1	
	Maximum	5	5	5	
In the evening, after teeth brushing, it is not allowed to eat, and only water can be drunk.	Median	5	5	5	0.060
	Minimum	4	1	1	
	Maximum	5	5	5	
The first permanent tooth erupts at the age of 6.	Median	5	5	5	0.577
	Minimum	1	1	1	
	Maximum	5	5	5	
Strong brushing is harmful to the teeth.	Median	5	5	4	0.005
	Minimum	1	1	1	
	Maximum	5	5	5	
The brush needs to be changed every 3 months.	Median	5	5	5	0.265
	Minimum	3	2	1	
	Maximum	5	5	5	

## Data Availability

The data that support the findings of this study are available from the corresponding author upon reasonable request.
